# Toenail Paronychium Flap: Novel Surgical Approach for Ingrowing Toenail and Review of the Literature of Conventional Surgical Methods

**DOI:** 10.1055/a-2040-0721

**Published:** 2023-05-29

**Authors:** Yujin Ahn, Hyunrok Lee, Surak Eo, Heakyeong Shin

**Affiliations:** 1 Department of Plastic and Reconstructive Surgery, College of Medicine, Dongguk University, Gyeongju, Republic of Korea; 2 Department of Plastic and Reconstructive Surgery, College of Medicine, Dongguk University, Ilsan, Republic of Korea

**Keywords:** ingrown toenail, nail flap surgery, paronychium flap

## Abstract

Ingrown toenails are most common among school-age children and adolescents though they can be observed at any age. Causes of ingrown toenails are increased curvature, trauma, and external pressure. Treatment of ingrown toenails can be broadly characterized as conservative and surgical. Conservative treatment can be performed using various methods, such as a gutter splint, dental floss, and cotton. Surgical treatments may be divided into two main approaches; narrowing of the nail plate and debulking of periungual tissues. However, these various conservative and surgical treatments have high recurrence rates, and thus, the author used a permanent surgical method based on the use of a paronychium flap to treat a 15-year-old male adolescent with excessive periungual tissues and curved ingrown toenails who did not improve despite conservative and several surgical treatments over 4 years. Subsequently, toenail shape was maintained without recurrence 22 months after surgery, and there were no complaints of inflammation or pain while walking. This simple surgical method can be performed on patients with advanced ingrown toenails due to excessive periungual tissues and nail curvature and can be expected to have permanent effects.

## Introduction

Ingrown toenails are most common among school-age children and adolescents though they can be encountered at any age. 1 The great toe is most commonly affected, especially the right great toe, which is subjected to greatest pressure when walking or running. 2 3 Causes of ingrown toenails include excessive skin surrounding the nail and excessive curvature (a hereditary factor), trauma, and external pressure (an acquired factor). These factors cause the edge of nail plate to dig into flesh, which causes pain and inflammation, and if not treated, symptoms gradually worsen and the formation of granulation tissue. 1 3 4

Treatment of ingrown toenails can be categorized as conservative or surgical. If symptoms are not severe, conservative treatment is performed using a gutter splint, dental floss, cotton wick, or band. However, if symptoms are severe or persist, surgical treatment is performed. 2 4 5 Nevertheless, high recurrence rates have been reported after surgery, and thus, the author attempted to devise a more permanent surgical method.

In the described case, local paronychium flaps were used to treat a patient who failed to improve after several conservative and surgical attempts over 4years. The method used has not been previously reported and is conducted to flatten toenails by redistributing periungual tissues and correcting the round shape of the nail bed, which was the root cause in the patient.

## Idea

**Fig. 1:**
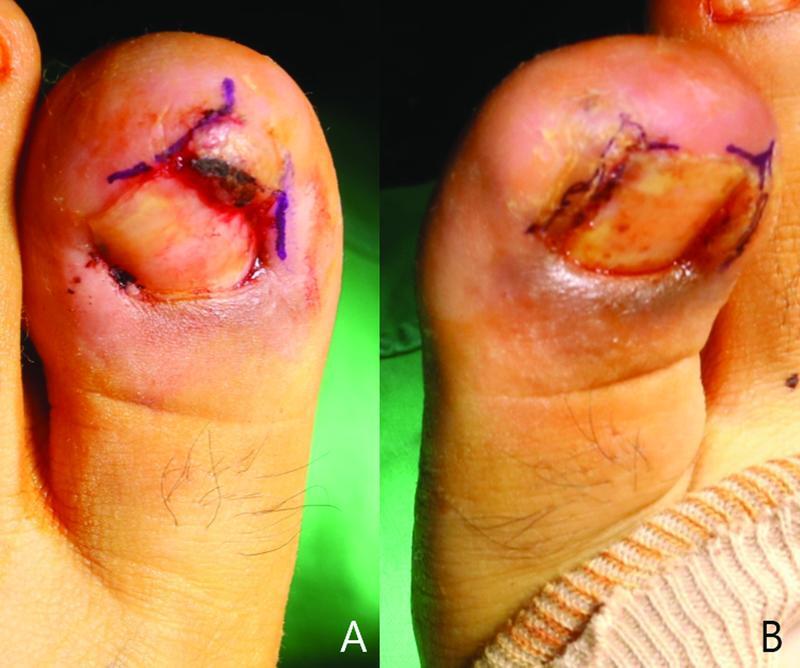
Preoperative photographs. The patient was 15 years old who had ingrown toenails on both sides digging into the flesh, causing severe inflammation and pain. ( **A** ) Left big toe. ( **B** ) Right big toe.

A 15-year-old male visited our clinic complaining of discomfort due to an ingrown toenail on both great toes ( Fig. 1 .). He complained of pain and inflammation during walking for 4 years and worsening symptoms while playing sports like soccer. He had been previously diagnosed with ingrown toenails at a local hospital, but after six surgeries and various conservative treatments over a 4-year period, the condition recurred several times, and symptoms did not improve. Inflammation due to ingrown toenails was also observed at admission and was accompanied by pain while walking. Therefore, we decided to perform local flap surgery using paronychium.

**Fig. 2:**
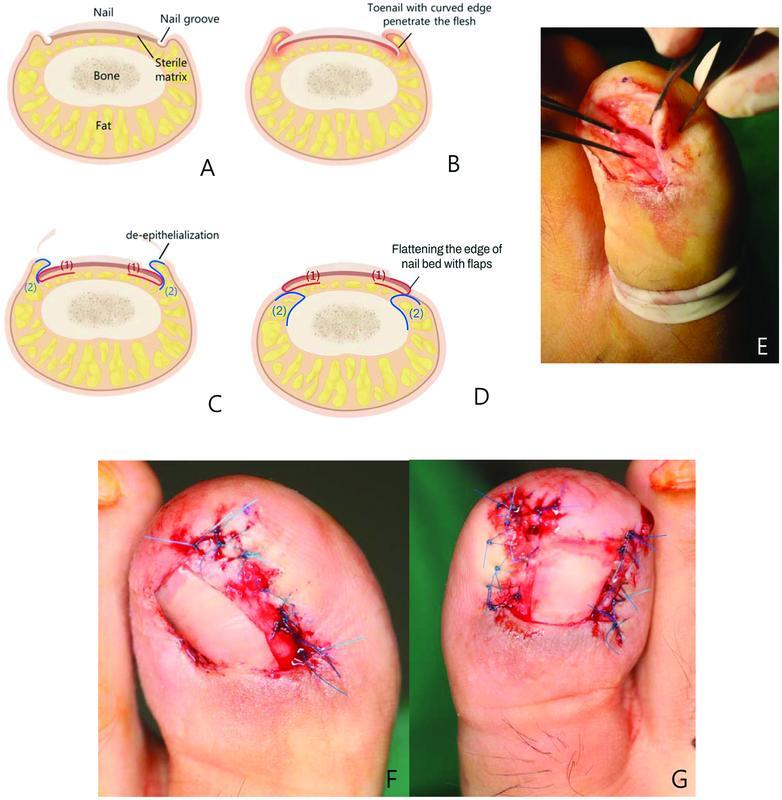
Schematic illustration and intraoperative photographs of paronychium flap technique. ( **A** ) Schematic illustration of normal toenail. ( **B** ) Ingrown toenail. ( **C** ) Deepithelialization of lateral nail folds were performed, adipose flaps of paronychium (2) and nail bed flaps (1) were elevated. ( **D** ) The separated paronychium adipose flap (2) was anchored to periosteum. Nail bed flaps (1) were then redraped over paronychium adipose flaps (2) and sutured to paronychium skin. ( **E** ) Intraoperative photographs of nail bed flap and paronychium skin flap were elevated over paronychium adipose flap. ( **F** , **G** ) Nail bed flaps were sutured to paronychium skin.

**Fig. 3:**
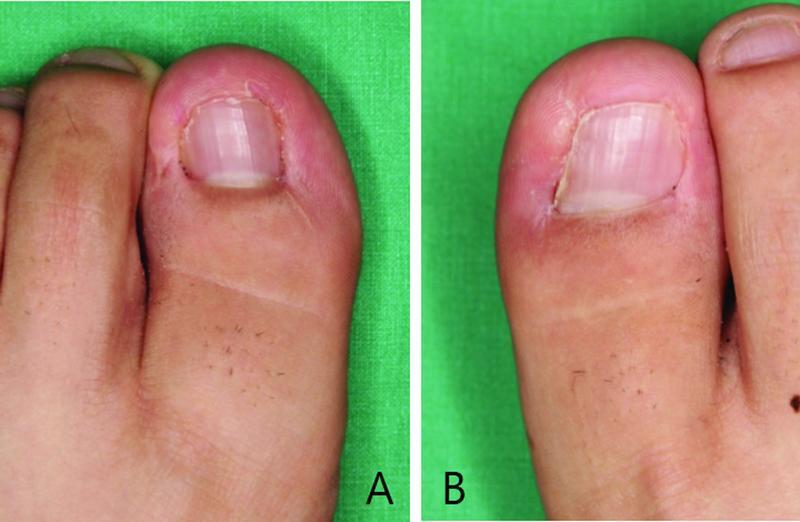
Postoperative photographs. 22 months' follow-up. Toenail shapes were well maintained without any deformation or sign of inflammation. ( **A** ) Left big toe. ( **B** ) Right big toe.

The procedure was conducted as follows. A 2% lidocaine solution was injected into the lateral nail folds of both great toes. Excision of granulation tissue and deepithelialization of lateral nail folds were performed. Incision was performed using a 15-blade scalpel at the lateral corner of the nail bed where the lateral nail fold meets the nail plate. When making an incision under the proximal nail fold, the incision was extended up to the most proximal part of the nail bed to allow full exposure of the lateral horn of the matrix. Blunt dissection using a freer elevator was then used to separate the vertically ingrown nail plate from the underlying nail bed, and a small part of the edge of the nail plate edge and the ingrowth were removed, followed by removal of the lateral matrix horn. After sharp dissection to separate the nail bed from the underlying periosteum at the lateral edge, a 15-blade scalpel was used to separate the adipose flap of paronychium from the dermal flap of paronychium, and the separated paronychium adipose flap was anchored to periosteum using absorbable 5–0 Vicryl. The nail bed flap was then redraped over the paronychium adipose flap and sutured to the paronychium dermal flap using absorbable 5–0 Vicryl and nonabsorbable 5–0 Nylon sutures ( Fig. 2 ). At 22 months postoperatively, toenail shapes were well maintained without any deformation or sign of inflammation ( Fig. 3 ).

## Discussion

Ingrown toenails have a variety of causes, and treatments vary accordingly. Causes include excess skin surrounding the nail, excessively rounded toenails, improperly trimmed nails, hyperhidrosis, poorly fitting footwear, trauma, subnail tumors, obesity, or excessive external pressure and cause the toenail border to irritate adjacent skin folds. During the early stage, the edge of the nail plate digs into flesh, causing pain and swelling, and as friction increases, oozing and infection. As the condition progresses, granulation tissue is created, and pain worsens, which makes it difficult to walk normally and greatly reduces quality of life. 3

Symptom severities can be roughly divided into three stages. In stage 1 ingrown toenails are characterized by erythema, slight edema, and pain. Stage 2 is characterized by symptom exacerbation, oozing, and infection, and in stage 3, symptoms are further aggravated, and hypertrophy of granulation tissue and lateral nail folds is evident. The present case was of stage 3. 2 3

Treatment of ingrown toenails may be conservative or surgical. Stage 1 cases are treated conservatively, and surgical treatment is considered from stage 2. However, conservative treatment is protracted, and cure rates are low, and thus, are often discontinued midway. On the other hand, surgery is performed simply under local anesthesia and can be highly effective. 3

Ingrown toenails can be treated using several procedures or variants of these procedures, which can be divided into two main approaches: narrowing of the nail plate or debulking of periungual tissues. 6 Wedge resection methods, introduced as Zadik's and Winograd's procedures, and chemical matricectomy, belong to the former approach. 5 In contrast, debulking methods involving excision of a soft tissue, introduced as Howard–Dubois or Super U procedures, and wide excision of excessive nail-fold granulation tissue, introduced as VandenBos' and Noël's procedures, belong to the latter approach. 6 7 8 9 Complications of wedge resection methods, like Zadik's procedure, include regrowth of a nail spicule secondary to incomplete matricectomy and postoperative nail bed infection. 10 11 Narrowing the nail surgically works but leaves a narrow unsightly nail. The main drawbacks of debulking methods are protracted healing and pain. Although, several surgical procedures have been introduced, recurrence rates are high for all procedures. For simple toenail removal, the recurrence rate is as high as 70%. 12 Zadik's operation with chemical ablation has a reported recurrence rate of 16 to 50%. 11

Debulking is directed toward reducing the volume of periungual tissues, whereas the toenail paronychium flap focuses on redistributing periungual tissues and making shallow nail folds, resulting in a flat nail bed and an excellent cosmetic outcome without pain. In the presented case, the patient had undergone several conservative treatments and six surgical treatments over 4 years but had experienced repeated recurrence. Thus, a paronychium flap was performed on a flat nail bed to redistribute periungual tissues and correct excessively curved toenails, and 22 months after surgery, toenail shape was maintained without discomfort or pain while walking. Therefore, we recommend this surgical method for treating advanced ingrown toenails due to excessive periungual tissues and nail curvature. A permanent curative effect can be expected after this simple operation.
